# Association of Antioxidants Use with All-Cause and Cause-Specific Mortality: A Prospective Study of the UK Biobank

**DOI:** 10.3390/antiox9121287

**Published:** 2020-12-16

**Authors:** Inken Behrendt, Gerrit Eichner, Mathias Fasshauer

**Affiliations:** 1 Institute of Nutritional Science, Justus-Liebig University of Giessen, 35390 Giessen, Germany; mathias.fasshauer@ernaehrung.uni-giessen.de; 2 Mathematical Institute, Justus-Liebig University of Giessen, 35392 Giessen, Germany; gerrit.eichner@math.uni-giessen.de; 3 Department of Internal Medicine (Endocrinology, Nephrology, and Rheumatology), University of Leipzig, 04103 Leipzig, Germany; 4 Leipzig University Medical Center, IFB AdiposityDiseases, 04103 Leipzig, Germany

**Keywords:** antioxidants, cancer, cardiovascular disease, dietary supplementation, metabolic syndrome, mortality, obesity, UK biobank

## Abstract

Prospective studies and randomized controlled trials elucidating the impact of antioxidants supplementation on mortality risk are inconclusive. The present analysis determined association between regular antioxidants use and all-cause (primary objective), as well as cause-specific, mortality in 345,626 participants of the UK Biobank cohort using Cox proportional hazard models. All models were adjusted for confounders and multiple testing. Antioxidants users were defined as participants who indicated to regularly use at least one of the following: multivitamins, vitamin C, vitamin E, selenium, and zinc. Median age of antioxidants users (*n* = 101,159) and non-users (*n* = 244,467) at baseline was 57 years. During 3.9 million person-years and a median follow-up of 11.5 years, 19,491 deaths occurred. Antioxidants use was not significantly associated with all-cause, cancer, and non-cancer mortality including several cancer and non-cancer subtypes. Interestingly, mortality risk from respiratory disease was significantly 21% lower among antioxidants users as compared to non-users (hazard ratio: 0.79; 95% confidence interval: 0.67, 0.92). In conclusion, the present study findings do not support recommendations for antioxidants supplementation to prevent all-cause, cancer, or non-cancer mortality on a population level. The significant inverse association between antioxidants use and respiratory disease mortality needs further study.

## 1. Introduction

High consumption of fruits and vegetables is part of a healthy eating pattern to prevent chronic diseases such as certain cancers and cardiovascular disease [1,2]. In 1990, the World Health Organization recommended an average intake of 400 g fruits and vegetables per day [2]. Several national scientific associations also propose sufficient consumption of fruits and vegetables. The National Health Service and the German Nutrition Association (DGE) suggest at least five portions of fruits and vegetables per day [3,4]. Furthermore, 2 cups of fruits and 2.5 cups of vegetables per day are proposed for people with daily energy requirements of 2000 kcal in the Dietary Guidelines for Americans 2015–2020 [5].

These recommendations are mainly based on observational epidemiological studies which reported an inverse association between the consumption of fruits and vegetables and several diseases [6]. In a recent meta-analysis of prospective studies, combined intake of fruits and vegetables was inversely associated with risk of cardiovascular disease, total cancer, and all-cause mortality [6]. Similar results were obtained when fruit and vegetable intakes were investigated separately [6]. Cardiovascular mortality risk decreased by 4% for each additional serving of fruits and vegetables per day in another meta-analysis of prospective studies [7].

The beneficial effects of fruits and vegetables consumption might be due to the high content of antioxidants [8]. Considering this hypothesis, antioxidant supplements including multivitamins, vitamin C, vitamin E, selenium, and zinc have been actively promoted to achieve similar protective effects in the prevention of cardiovascular disease and cancer for decades. However, results of randomized clinical trials examining the association between antioxidants supplementation and metabolic, as well as vascular, health have been heterogeneous ranging from beneficial to harmful effects [9]. In 2013, the US Preventive Services Task Force declared that the current evidence is inadequate to recommend dietary supplementation of antioxidant vitamins, multivitamins, or antioxidant combinations for preventing cardiovascular disease or cancer [9]. Similarly, several scientific associations recommend the intake of vitamins and minerals from a varied and balanced diet rather than from dietary supplements [10,11]. In addition, dietary supplementation is not recommended for healthy people who are on a balanced diet [12]. Furthermore, the DGE notes that dietary supplements are unsuitable to compensate unfavorable dietary habits [12].

Despite these recommendations, the use of dietary supplements is increasing [13]. In the UK, the dietary supplements market increased from £670 million in 2009 [10] to £906 million in 2016 [14]. The global dietary supplement market was estimated at 123 billion USD in 2019 and is expected to increase up to 230 billion USD in 2027 [15]. The Food Standards Agency recently reported that almost 50% of UK adults regularly take food supplements [14]. Multivitamins were consumed most frequently followed by fish oil and vitamin C [14].

Most prospective observational studies examining the association between antioxidants supplementation and mortality are restricted to a narrow range of death causes and focus on effects of single vitamins, minerals, or multivitamins. Furthermore, a possible link between antioxidants use and major mortality causes including pancreas cancer and respiratory disease has not been studied so far. To address these limitations, the current study assesses the association of antioxidant supplements with a broad range of death causes in a large, well-characterized UK cohort. It is hypothesized that antioxidants supplementation is associated with decreased all-cause, as well as cause-specific, mortality risk.

## 2. Materials and Methods

### 2.1. Study and Participants

UK Biobank is a large multicenter, prospective cohort study [16]. A detailed study protocol is available at https://www.ukbiobank.ac.uk. In brief, more than 500,000 participants provided information regarding lifestyle, socioeconomic status, health conditions, and medication and dietary supplement use at baseline (2006–2010). In addition, physical measurements were conducted at 22 assessment centers during the baseline visit. Participants with missing data for dietary supplement use and confounding factors, i.e., smoking status, alcohol intake, physical activity, total household income, qualifications, ethnic background, and percentage body fat, were excluded. Women with suspected or confirmed pregnancy were also excluded from the analyses (Figure S1). UK Biobank obtained permission by the North West Multicentre Research Ethics Committee and all participants gave written informed consent to participate in the study [16].

### 2.2. Exposure Assessment

Dietary supplement use was self-reported. Participants conducted a touchscreen questionnaire during their baseline assessment with two questions addressing regular use of dietary supplements. Participants who indicated to use at least one of the following dietary supplements were classified as antioxidants users: multivitamins, vitamin C, vitamin E, selenium, and zinc.

### 2.3. Outcome Assessment

Mortality data including the date and underlying primary cause of death were obtained for each participant through linkage to the NHS Information Centre for participants from England and Wales and by NHS Central Register, Scotland for participants from Scotland [17]. Participants were followed up from the date of their baseline visit to the date of death, loss to follow-up, or the censor date (31 August 2020) whichever came first. According to the *International Statistical Classification of Diseases and Related Health Problems, Tenth Revision* (ICD-10) codes, the following two main mortality categories were defined: cancer (C00-D48) and non-cancer (all apart from C00-D48). Within cancer mortality, colorectal cancer was categorized as C18–C20, bronchus and lung cancer as C34, female breast cancer as C50, pancreatic cancer as C25, and prostate cancer as C61. Within non-cancer mortality, cardiovascular disease (I00–I79), respiratory disease (J09–J18 and J40–J47), ischemic heart disease (I20–I25), and stroke (I60–I69) were analyzed.

### 2.4. Statistical Analyses

Statistical analyses have been described recently [18,19]. In brief, the statistical software R version 3.6.3 [20] together with add-on packages was used to analyze the data. Baseline characteristics of antioxidants users and non-users were compared using chi-square test for categorical variables and Mann–Whitney U test for continuous variables. Cox proportional hazard models were used to calculate hazard ratios (HRs) and 95% confidence intervals (CIs) for associations of antioxidants use and all-cause, as well as cause-specific, mortality. The proportional-hazard assumption was tested using Schoenfeld residuals. If a covariate violated this assumption, a stratification of this covariate was included in the final model. Models were adjusted for potential risk factors, i.e., age, sex, smoking status, alcohol intake, total household income per year, qualifications, ethnic background, total physical activity, and percentage body fat. Holm’s method was used to adjust for multiple testing. The significance level was set at *p* < 0.05.

## 3. Results

### 3.1. Baseline Characteristics of UK Biobank Participants

Median (Q1, Q3) age of the study population was 57 (49, 63) years with 51.1% of participants being female. Among 345,626 participants included in the analyses, 101,159 (29.3%) were regular antioxidant supplement users. Detailed baseline characteristics of the study population by antioxidants use are presented in Table 1. During 3.9 million person-years and a median follow-up of 11.5 years, 19,491 deaths occurred. There were 10,780 deaths from cancer (55.3%) and 8711 deaths from non-cancer (44.7%). A summary statistic for the numbers of deaths from cancer and non-cancer subtypes is given in Table 2.

### 3.2. Antioxidants Use and All-Cause Mortality

Regular supplementation of antioxidant vitamins and minerals was inversely associated with all-cause mortality (HR: 0.92; 95% CI: 0.89, 0.95, *p* < 0.0001). However, this association was no longer statistically significant after adjusting for age, sex, smoking status, alcohol intake, total household income, qualifications, ethnic background, physical activity, and percentage body fat (HR: 0.97; 95% CI: 0.94, 1.01, *p* = 0.0977).

### 3.3. Antioxidants Use and Cause-Specific Mortality

Antioxidants use was associated with neither total cancer mortality (HR: 0.99; 95% CI: 0.95, 1.03) nor deaths from any of the investigated most common cancer subtypes, i.e., colorectal, bronchus and lung, female breast, pancreatic, and prostate cancer (Table 2). In addition, regular supplementation of antioxidant vitamins and minerals was not significantly associated with non-cancer mortality after adjusting for confounders (HR: 0.95; 95% CI: 0.91, 1.00; Table 2). Within non-cancer subtypes, antioxidants use was significantly associated with a decreased mortality risk from cardiovascular disease (HR: 0.92; 95% CI: 0.86, 0.99) and ischemic heart disease (HR: 0.87; 95% CI: 0.79, 0.97) (Table 2). However, these associations did not remain statistically significant after adjustment for multiple testing (Table 2). There was no significant association between antioxidants use and stroke mortality (Table 2). Interestingly, dietary supplementation of antioxidants was associated with a 21% lower respiratory disease mortality risk (HR: 0.79; 95% CI: 0.67, 0.92), a finding which remained statistically significant after adjusting for multiple comparisons (Table 2). Association between antioxidants use and mortality risk from respiratory disease remained statistically significant in a sensitivity analysis excluding participants who died or were lost to follow-up within two years after baseline (HR: 0.80; 95% CI: 0.68, 0.93, *p* = 0.0053).

## 4. Discussion

In the current study, antioxidants use is not significantly related to overall, cancer, and non-cancer mortality in a large, well-characterized European cohort. However, it is shown for the first time that supplementation of antioxidant vitamins and minerals is inversely associated with respiratory disease mortality.

The results of the present study on overall mortality support recent evidence from the National Health and Nutrition Examination Survey [21]. The authors demonstrate convincingly that multivitamins, vitamin C, vitamin E, selenium, or zinc supplementation is not significantly associated with all-cause mortality after adjusting for confounders [21]. Similarly, any vitamin or mineral use is not significantly related to all-cause mortality in two prospective cohort studies [22,23]. In agreement with these findings, single supplementation of multivitamins [21,22,24,25,26,27], vitamin C [21,26,28,29,30], vitamin E [21,24,28,29,30,31], selenium [21,28], or zinc [21,28] is not linked with all-cause mortality risk. In contrast, long-term single supplementation of vitamin C and vitamin E is related to a small decrease in mortality risk in the Vitamin and Lifestyle Study [25]. Supplementation of vitamin E in combination with vitamin C but not single use of vitamin C or vitamin E is associated with a decreased mortality risk in an elderly population [26]. All-cause-mortality risk is significantly reduced by supplementation of antioxidant vitamins including vitamin A, C, or E and their combinations in the European Prospective Investigation into Cancer and Nutrition (EPIC)-Heidelberg cohort [22]. Use of vitamin A, C, or E alone or in combination with multivitamins is related to reduced all-cause mortality risk in the Cancer and Prevention Study II, whereas multivitamin supplementation alone shows an opposite trend [32]. In agreement with the latter finding, long-term multivitamin supplementation is associated with an increased mortality risk in postmenopausal women [28]. In a recent meta-analysis including 21 randomized clinical trials, supplementation of at least two antioxidants, i.e., vitamin A, vitamin C, vitamin E, β-carotene, selenium, or zinc, is associated with a slightly increased all-cause mortality risk [33]. Taking these published studies and the current evidence into consideration, antioxidants do not show consistent beneficial effects on all-cause mortality.

In the current study, overall cancer mortality risk is not affected by baseline supplementation of antioxidants. This null finding is in line with most studies examining the association between single dietary supplementation of multivitamins [21,22,23,25,26,28,34], vitamin C [21,23,26,28], vitamin E [21,23,25,26,28], selenium [21,28], or zinc [21,28] and cancer mortality. Furthermore, non-specified supplement use is not associated with cancer mortality in a cohort of Swedish men [23]. Similarly, cancer mortality risk is not affected by any vitamin or mineral use at baseline in a German EPIC-cohort [22]. Meta-analyses of randomized clinical trials show no beneficial effects of single and combined antioxidants use for primary or secondary prevention of cancer, as well as cancer mortality [35,36]. Supplemental vitamin C intake is associated with decreased cancer mortality risk in a prospective US population study [25]. Furthermore, supplementation of vitamin A, C, or E and their combinations is inversely associated with cancer mortality risk [22]. To the best of our knowledge, association between antioxidants use on the one hand and cancer separated into major subtypes on the other hand within one study has only been analyzed so far in one epidemiological cohort. Watkins and co-workers demonstrated that mortality risk from colon, lung, female breast, and prostate cancer is comparable or higher among participants with history of cancer taking antioxidant vitamins as compared to non-users [32]. However, these associations are no longer statistically significant after excluding deaths in the first three years after baseline [32]. In accordance with the latter analysis, antioxidants use is not significantly associated with colorectal, bronchus and lung, female breast, and prostate cancer mortality in the current study. Furthermore, antioxidants supplementation is not linked to pancreas cancer mortality in the present report. To the best of our knowledge, association between antioxidants use and pancreas cancer has not been assessed so far in a large epidemiological study. Published evidence and the current findings do not support the hypothesis that a relevant association exists between antioxidants use and mortality risk from cancer and major cancer subtypes.

In the present report, regular use of antioxidant vitamins and minerals is not significantly associated with non-cancer mortality. Interestingly, antioxidants supplementation is associated with significantly reduced all-cause and non-cancer mortality risk in participants ≥60 but not <60 years of age (Table S1). In contrast to these findings, association between multivitamin use and all-cause, cancer, and cardiovascular mortality risk is not modified by age in another prospective study [34]. Mortality from cardiovascular disease and ischemic heart disease but not stroke is significantly lower in supplement users as compared to non-users in the present study. However, all significant associations are attenuated to non-significant levels after adjusting for multiple comparisons. Findings of other prospective studies are contradictory, with most studies showing no association between single use of multivitamins [21,22,24,28,37], vitamin C [21,25,28,37], vitamin E [21,24,28,37], selenium [21,28], or zinc [21,28] and cardiovascular mortality. Furthermore, single multivitamin [27,32,38], vitamin C [39], and vitamin E [39] use does not show any association with stroke mortality. In agreement with these reports, single or combined supplementation of antioxidants has no effect on cardiovascular, ischemic heart disease, and stroke mortality in a recent systematic review and meta-analysis of randomized controlled trials [33]. However, lower mortality from cardiovascular disease [25,26] and ischemic heart disease [32] has also been observed in some studies on antioxidant supplements. To the best of our knowledge, the association between supplementation of antioxidants and respiratory disease mortality is examined for the first time in the present study. Interestingly, users of antioxidant vitamins and minerals have a 21% lower respiratory disease mortality risk as compared to non-users. This association remains statistically significant after adjusting for multiple testing and in a sensitivity analysis excluding participants who died or were lost to follow-up within two years after baseline. Taking published studies and the current analysis into consideration, a relevant association between antioxidants use and non-cancer mortality is unlikely. The significant associations between antioxidants supplementation and respiratory disease mortality, as well as all-cause and non-cancer mortality in participants ≥ 60 years of age, need further analyses.

This study has several strengths. First, UK Biobank is a large and well-characterized cohort. The present analysis includes more than 345,000 participants which enables some analyses for the first time. Second, recall bias is reduced due to the prospective design of the study. Third, adjustment for several potential confounders is possible due to the well-characterized study cohort. However, results should be interpreted in the context of several limitations. There was no information about the duration of antioxidants use and the dosage of dietary supplements so that a dose–response relationship cannot be assessed. Furthermore, incident health conditions during follow-up might influence supplementation behavior and confound the association between antioxidant vitamins and minerals supplementation and mortality risk. In addition, no information is available in the present study concerning cancer etiopathogenesis, i.e., hereditary versus idiopathic cancer. Association between antioxidants use and cancer mortality might well depend on cancer etiopathogenesis since recent studies suggest a protective role of antioxidants in preventing hereditary cancers [40,41]. This hypothesis should be addressed in future studies.

## 5. Conclusions

All-cause, cancer, and non-cancer mortality is not associated with antioxidants use in a large epidemiological cohort. It is unlikely that a population in total benefits from supplementation of antioxidant vitamins and minerals. The negative associations between antioxidants use and respiratory disease mortality, as well as all-cause and non-cancer mortality in participants ≥60 years of age, need further study.

## Figures and Tables

**Table 1:** Baseline characteristics of the UK Biobank cohort by antioxidants use. ^1^

Characteristics	Overall (*n* = 345,626)	Antioxidants Non-User (*n* = 244,467)	Antioxidants User (*n* = 101,159)	*p*-Value
**Age** (years)	57 (49, 63)	57 (49, 63)	57 (50, 63)	<0.0001
**Female**	176,602 (51.1)	118,746 (48.6)	57,856 (57.2)	<0.0001
**Smoking status**	-	-	-	<0.0001
Never	188,832 (54.6)	133,514 (54.6)	55,318 (54.7)	-
Previous	121,388 (35.1)	85,153 (34.8)	36,235 (35.8)	-
Current	35,406 (10.2)	25,800 (10.6)	9606 (9.5)	-
**Alcohol intake**	-	-	-	<0.0001
Never	23,705 (6.9)	16,346 (6.7)	7359 (7.3)	-
Special occasions only	35,425 (10.3)	24,314 (9.9)	11,111 (11.0)	-
One to three times a month	37,817 (10.9)	26,472 (10.8)	11,345 (11.2)	-
Once or twice a week	88,607 (25.6)	62,630 (25.6)	25,977 (25.7)	-
Three to four times a week	84,451 (24.4)	60,319 (24.7)	24,132 (23.9)	-
Daily or almost daily	75,621 (21.9)	54,386 (22.2)	21,235 (21.0)	-
**Total household income per year (£)**	-	-	-	<0.0001
<18,000	72,014 (20.8)	51,015 (20.9)	20,999 (20.8)	-
18,000–30,999	86,006 (24.9)	60,141 (24.6)	25,865 (25.6)	-
31,000–51,999	92,051 (26.6)	65,120 (26.6)	26,931 (26.6)	-
52,000–99,999	74,984 (21.7)	53,479 (21.9)	21,505 (21.3)	-
≥100,000	20,571 (6.0)	14,712 (6.0)	5859 (5.8)	-
**Qualifications**	-	-	-	<0.0001
Other	17,153 (5.0)	12,022 (4.9)	5131 (5.1)	-
NVQ or HND or HNC equivalent	22,590 (6.5)	16,261 (6.7)	6329 (6.3)	-
CSEs or equivalent	17,736 (5.1)	13,031 (5.3)	4705 (4.7)	-
O levels/GCSEs or equivalent	73,121 (21.2)	51,652 (21.1)	21,469 (21.2)	-
A levels/AS levels or equivalent	41,584 (12.0)	28,744 (11.8)	12,840 (12.7)	-
College or university degree	128,271 (37.1)	89,265 (36.5)	39,006 (38.6)	-
Noa	45,171 (13.1)	33,492 (13.7)	11,679 (11.5)	-
**Ethnic background**	-	-	-	<0.0001
White	329,823 (95.4)	234,403 (95.9)	95,420 (94.3)	-
Non-White	15,803 (4.6)	10,064 (4.1)	5739 (5.7)	-
**Total physical activity** (MET-min/week)	1760 (809, 3520)	1710 (773, 3451)	1878 (889, 3666)	<0.0001
**Percentage body fat**	30.3 (24.6, 36.9)	30.0 (24.5, 36.7)	30.8 (25.0, 37.3)	<0.0001

^1^ Categorical variables are presented as number (percentage) and continuous variables as median (Q1, Q3). Abbreviations: CSE, Certificate of Secondary Education; GCSE, General Certificate of Secondary Education; HNC, Higher National Certificate; HND, Higher National Diploma; MET, Metabolic equivalent of task; Noa, None of the above; NVQ, National Vocational Qualification; Other, Other professional qualifications, e.g., nursing and teaching; Q, Quartile.

**Table 2:** Association between regular antioxidants use (independent variable) and cause-specific mortality (*n* = 345,626). ^1^

Cause of Death	Events	HR	95% CI	*p*-Value	Holm-Adjusted *p*-Value
**Cancer**	10,780	-	-	-	-
Non-users	7706	1.00	-	-	-
Users	3074	0.99	(0.95, 1.03)	0.6152	1.0000
**Non-cancer**	8711	-	-	-	-
Non-users	6396	1.00	-	-	-
Users	2315	0.95	(0.91, 1.00)	0.0559	0.4471
**Cancer subtypes:**	-	-	-	-	-
**Colorectal**	1074	-	-	-	-
Non-users	793	1.00	-	-	-
Users	281	0.89	(0.78, 1.02)	0.0896	0.6269
**Bronchus and lung**	1726	-	-	-	-
Non-users	1275	1.00	-	-	-
Users	451	0.92	(0.82, 1.02)	0.1199	0.7197
**Female breast ^2^**	828	-	-	-	-
Non-users	543	1.00	-	-	-
Users	285	1.07	(0.92, 1.23)	0.3848	1.0000
**Pancreatic**	783	-	-	-	-
Non-users	551	1.00	-	-	-
Users	232	1.04	(0.89, 1.22)	0.5950	1.0000
**Prostate ^3^**	702	-	-	-	-
Non-users	503	1.00	-	-	-
Users	199	1.11	(0.94, 1.31)	0.2260	1.0000
**Non-cancer subtypes:**	-	-	-	-	-
**Cardiovascular disease**	3701	-	-	-	-
Non-users	2756	1.00	-	-	-
Users	945	0.92	(0.86, 0.99)	**0.0317**	0.2854
**Ischemic heart disease**	2076	-	-	-	-
Non-Users	1586	1.00	-	-	-
Users	490	0.87	(0.79, 0.97)	**0.0096**	0.0960
**Stroke**	729	-	-	-	-
Non-users	521	1.00	-	-	-
Users	208	0.97	(0.83, 1.15)	0.7466	1.0000
**Respiratory disease**	901	-	-	-	-
Non-users	697	1.00	-	-	-
Users	204	0.79	(0.67, 0.92)	**0.0028**	0.0310

^1^ P for all models overall: <0.0001. All models were adjusted for age, sex, smoking status, alcohol intake, income, qualifications, ethnic background, physical activity, and percentage body fat. ^2^ Female only (*n* = 176,602). ^3^ Male only (*n* = 169,024). Abbreviations: CI, Confidence interval; HR, Hazard ratio.

## Supplementary Materials

The following are available online at https://www.mdpi.com/2076-3921/9/12/1287/s1, Figure S1: Venn diagram depicting the number of participants excluded by five exclusion criteria; Table S1: Association between regular antioxidants use (independent variable) and cause-specific mortality in participants <60 years (*n* = 206,586) and ≥60 years (*n* = 139,040) of age.

## References

[1] Dauchet L., Amouyel P., Hercberg S., Dallongeville J. (2006). Fruit and vegetable consumption and risk fruit and vegetable consumption and risk of coronary heart disease: A meta-analysis of cohort studies. J. Nutr..

[2] WHO (1990). Diet, Nutrition and the Prevention of Chronic Diseases.

[3] NHS Why 5 a Day?. https://www.nhs.uk/live-well/eat-well/why-5-a-day/.

[4] DGE Fruit and Vegetables. It’s the Quantity. https://www.dge.de/wissenschaft/weitere-publikationen/fachinformationen/obst-und-gemuese-die-menge-machts/.

[5] U.S. Department of Health and Human Services, U.S. Department of Agriculture 2015–2020 Dietary Guidelines for Americans, 8th ed. http://health.gov/dietaryguidelines/2015/guidelines/.

[6] Aune D., Giovannucci E., Boffetta P., Fadnes L.T., Keum N., Norat T., Greenwood D.C., Riboli E., Vatten L.J., Tonstad S. (2017). Fruit and vegetable intake and the risk of cardiovascular disease, total cancer and all-cause mortality—A systematic review and dose-response meta-analysis of prospective studies. Int. J. Epidemiol..

[7] Wang X., Ouyang Y., Liu J., Zhu M., Zhao G., Bao W., Hu F.B. (2014). Fruit and vegetable consumption and mortality from all causes, cardiovascular disease, and cancer: Systematic review and dose-response meta-analysis of prospective cohort studies. BMJ.

[8] Hertog M.G.L., Bueno-de-Mesquita H.B., Fehily A.M., Sweetman P.M., Elwood P.C., Kromhout D. (1996). Fruit and vegetable consumption and cancer mortality in the caerphilly study. Cancer Epidemiol. Biomark. Prevent..

[9] Fortmann S.P., Burda B.U., Senger C.A., Lin J.S., Whitlock E.P. (2013). Vitamin and mineral supplements in the primary prevention of vitamin and mineral supplements in the primary prevention of cardiovascular disease and cancer: An updated systematic evidence review for the U.S. preventive services task force. Ann. Intern. Med..

[10] NHS Supplements. Who Needs Them? A Behind the Headlines Report. https://www.nhs.uk/news/2011/05May/Documents/BtH_supplements.pdf.

[11] Marra M.V., Boyar A.P. (2009). Position of the American Dietetic Association: Nutrient supplementation. J. Am. Diet. Assoc..

[12] Bechthold A., Albrecht V., Leschik-Bonnet E., Heseker H. (2012). DGE statement: Evaluation of vitamin supplies in Germany, part 2: Critical vitamins and vitamin supplies in special situations. Ernähr. Umsch..

[13] Skeie G., Braaten T., Hjartåker A., Lentjes M., Amiano P., Jakszyn P., Pala V., Palanca A., Niekerk E.M., Verhagen H. (2009). Use of dietary supplements in the European Prospective Investigation into cancer and nutrition calibration study. Eur. J. Clin. Nutr..

[14] Food Standards Agency Food Supplements Consumer Research. https://www.food.gov.uk/sites/default/files/media/document/food-supplements-consumer-research.pdf.

[15] Market Analysis Report. Dietary Supplements Market Size, Share & Trends Analysis Report by Ingredient (Vitamins, Minerals), by Form, by Application, by End User, by Distribution Channel, by Region, and Segment Forecasts, 2020–2027. https://www.grandviewresearch.com/industry-analysis/dietary-supplements-market.

[16] UK Biobank Protocol for a Large-Scale Prospective Epidemiological Resource.

[17] UK Biobank Mortality Data: Linkage from National Death Registries.

[18] Behrendt I., Fasshauer M., Eichner G. (2020). Gluten intake and all-cause and cause-specific mortality: Prospective findings from the UK Biobank. J. Nutr..

[19] Behrendt I., Fasshauer M., Eichner G. (2020). Gluten intake and metabolic health: Conflicting findings from the UK Biobank. Eur. J. Nutr..

[20] R Core Team R: A Language and Environment for Statistical Computing. https://www.R-project.org/.

[21] Chen F., Du M., Blumberg J.B., Ho Chui K.K., Ruan M., Rogers G., Shan Z., Zeng L., Zhang F.F. (2019). Association among dietary supplement use, nutrient intake, and mortality among U.S. adults: A cohort study. Ann. Intern. Med..

[22] Li K., Kaaks R., Linseisen J., Rohrmann S. (2012). Vitamin/mineral supplementation and cancer, cardiovascular, and all-cause mortality in a German prospective cohort (EPIC-Heidelberg). Eur. J. Nutr..

[23] Messerer M., Håkansson N., Wolk A., Akesson A. (2008). Dietary supplement use and mortality in a cohort of Swedish men. Br. J. Nutr..

[24] Stampfer M.J., Hennekens C.H., Manson J.E., Colditz G.A., Rosner B., Willett W.C. (1993). Vitamin E consumption and the risk of coronary disease in women. N. Engl. J. Med..

[25] Pocobelli G., Peters U., Kristal A.R., White E. (2009). Use of supplements of multivitamins, vitamin C, and vitamin E in relation to mortality. Am. J. Epidemiol..

[26] Losonczy K.G., Harris T.B., Havlik R.J. (1996). Vitamin E and vitamin C supplement use and risk of all-cause and coronary heart disease mortality in older persons: The established populations for epidemilogic studies of their elderly. Am. J. Clin. Nutr..

[27] Neuhouser M.L., Wassertheil-Smoller S., Thomson C., Aragaki A., Anderson G.L., Manson J.E., Patterson R.E., Rohan T.E., van Horn L., Shikany J.M. (2009). Multivitamin use and risk of cancer and cardiovascular disease in the women’s health initiative cohorts. Arch. Intern. Med..

[28] Mursu J., Robien K., Harnack L.J., Park K., Jacobs D.R. (2011). Dietary supplements and mortality rate in older women: The Iowa women’s health study. Arch. Intern. Med..

[29] Roswall N., Olsen A., Christensen J., Hansen L., Dragsted L.O., Overvad K., Tjønneland A. (2012). Micronutrient intake in relation to all-cause mortality in a prospective Danish cohort. Food Nutr. Res..

[30] Brzozowska A., Kaluza J., Knoops K.T.B., de Groot L.C.P.G.M. (2008). Supplement use and mortality: The SENECA study. Eur. J. Nutr..

[31] Hayden K.M., Welsh-Bohmer K.A., Wengreen H.J., Zandi P.P., Lyketsos C.G., Breitner J.C.S. (2007). Risk of mortality with vitamin E supplements: The Cache county study. Am. J. Med..

[32] Watkins M.L., Erickson J.D., Thu M.J., Mulinare J., Heath C.W. (2000). Multivitamin use and mortality in a large prospective study. Am. J. Epidemiol..

[33] Jenkins D.J.A., Spence J.D., Giovannucci E.L., Kim Y.-I., Josse R., Vieth R., Blanco Mejia S., Viguiliouk E., Nishi S., Sahye-Pudaruth S. (2018). Supplemental vitamins and minerals for CVD prevention and treatment. J. Am. Coll. Cardiol..

[34] Park S.-Y., Murphy S.P., Wilkens L.R., Henderson B.E., Kolonel L.N. (2011). Multivitamin use and the risk of mortality and cancer incidence: The multiethnic cohort study. Am. J. Epidemiol..

[35] Myung S.-K., Kim Y., Ju W., Choi H.J., Bae W.K. (2010). Effects of antioxidant supplements on cancer prevention: Meta-analysis of randomized controlled trials. Ann. Oncol..

[36] Schwingshackl L., Boeing H., Stelmach-Mardas M., Gottschald M., Dietrich S., Hoffmann G., Chaimani A. (2017). Dietary supplements and risk of cause-specific death, cardiovascular disease, and cancer: A systematic review and meta-analysis of primary prevention trials. Adv. Nutr..

[37] Muntwyler J., Hennekens C.H., Manson J.E., Buring J.E., Gaziano M.J. (2002). Vitamin supplement use in a low-risk population of U.S. male physicians and subsequent cardiovascular mortality. Arch. Intern. Med..

[38] Adebamowo S.N., Feskanich D., Stampfer M., Rexrode K., Willett W.C. (2017). Multivitamin use and risk of stroke incidence and mortality amongst women. Eur. J. Neurol..

[39] Yochum L.A., Folsom A.R., Kushi L.H. (2000). Intake of antioxidant vitamins and risk of death from stroke in postmenopausal women. Am. J. Clin. Nutr..

[40] Kotsopoulos J., Narod S.A. (2005). Towards a dietary prevention of hereditary breast cancer. Cancer Causes Control.

[41] Li M., Chen Q., Ma T., Yu X. (2017). Targeting reactive nitrogen species suppresses hereditary pancreatic cancer. Proc. Natl. Acad. Sci. USA.

