# Supplementary material for: Association of Antioxidants Use with All-Cause and Cause-Specific Mortality: A Prospective Study of the UK Biobank
Source: Antioxidants (Basel). 2020 Dec 16;9(12):1287. doi: 10.3390/antiox9121287 (PMC7766648; doi:10.3390/antiox9121287)
Supplement: Supplementary file 1 [file antioxidants-09-01287-s001.pdf]

Supplementary Material

Antioxidants

Supplementation of antioxidants is not associated with all-cause and cause-specific mortality risk: A prospective study of the UK Biobank

Authors: Inken Behrendt[1,*], Gerrit Eichner[2,#] and Mathias Fasshauer[1,3,4,#]

[1] Institute of Nutritional Science, Justus-Liebig University of Giessen, Giessen, Germany inken.behrendt@ernaehrung.uni-giessen.de (I.B.) mathias.fasshauer@ernaehrung.uni-giessen.de (M.F.)
[2] Mathematical Institute, Justus-Liebig University of Giessen, Giessen, Germany gerrit.eichner@math.uni-giessen.de
[3] Department of Internal Medicine (Endocrinology, Nephrology, and Rheumatology), University of Leipzig, Leipzig, Germany
[4] Leipzig University Medical Center, IFB AdiposityDiseases, Leipzig, Germany
* Correspondence: inken.behrendt@ernaehrung.uni-giessen.de
\# These authors equally contributed to this work

#These authors contributed equally to this work.
*Corresponding author. Mailing address: Goethestr. 55, 35390 Giessen, Germany. Phone: +49 641 9939067. Fax: +49 641 9939069. E-mail: inken.behrendt@ernaehrung.uni-giessen.de.

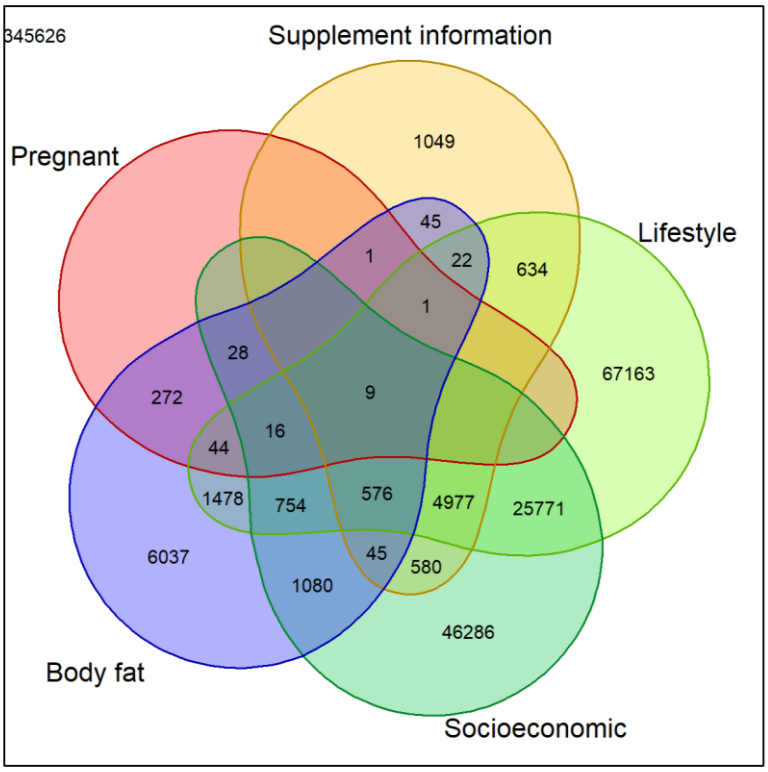

Figure S1 Venn diagram depicting the number of participants excluded by five exclusion criteria

Table S1 Association between regular antioxidants use (independent variable) and cause-specific mortality in participant <60 years (n = 206,586) and ≥60 years (n = 139,040) of age.[1]

Cause of death	Events	HR	95% CI	*p*-value[1]
**< 60 years of age**				
**All-cause**	6032	-	-	-
Non-users	4360	1.00	-	-
Users	1672	1.01	(0.95, 1.07)	0.7157
**Cancer**	3506	-	-	-
Non-users	2521	1.00	-	-
Users	985	0.99	(0.92, 1.06)	0.7164
**Non-cancer**	2526	-	-	-
Non-users	1839	1.00	-	-
Users	687	1.05	(0.96, 1.15)	0.2793
**≥ 60 years of age**				
**All-cause**	13,459	-	-	-
Non-users	9742	1.00	-	-
Users	3717	0.96	(0.92, 0.99)	**0.0234**
**Cancer**	7274	-	-	-
Non-users	5185	1.00	-	-
Users	2089	0.99	(0.94, 1.04)	0.6976
**Non-cancer**	6185	-	-	-
Non-users	4557	1.00	-	-
Users	1628	0.92	(0.87, 0.97)	**0.0034**

[1]P for all models overall: <0.0001. All models were adjusted for age, sex, smoking status, alcohol intake, income, qualifications, ethnic background, physical activity, and percentage body fat.
Abbreviations: CI, Confidence interval; HR, Hazard ratio.
